# Nanoconfined Chlorine-Substituted Monomethine Cyanine Dye with a Propionamide Function Based on the Thiazole Orange Scaffold—Use of a Fluorogenic Probe for Cell Staining and Nucleic Acid Visualization

**DOI:** 10.3390/molecules29246038

**Published:** 2024-12-21

**Authors:** Nikolay Ishkitiev, Maria Micheva, Marina Miteva, Stefaniya Gaydarova, Christo Tzachev, Vesela Lozanova, Valentin Lozanov, Diana Cheshmedzhieva, Meglena Kandinska, Sonia Ilieva, Raimundo Gargallo, Stanislav Baluschev, Stoyno Stoynov, Teodora Dyankova-Danovska, Marina Nedelcheva-Veleva, Katharina Landfester, Zornitsa Mihaylova, Aleksey Vasilev

**Affiliations:** 1 Department of Medical Chemistry and Biochemistry, Medical University Sofia, 2 Zdrave Str., 1431 Sofia, Bulgaria; nishkitiev@medfac.mu-sofia.bg (N.I.); mia9580@yahoo.com (M.M.); vlozanova@medfac.mu-sofia.bg (V.L.); vlozanov@medfac.mu-sofia.bg (V.L.); 2 Max Planck Institute for Polymer Research, Ackermannweg 10, 55128 Mainz, Germany; michevam@mpip-mainz.mpg.de (M.M.); balouche@mpip-mainz.mpg.de (S.B.); landfester@mpip-mainz.mpg.de (K.L.); 3 Faculty of Chemistry and Pharmacy, Sofia University “St. Kliment Ohridski”, 1 James Bourchier Blvd., 1164 Sofia, Bulgaria; stefaniya.gaydarova@gmail.com (S.G.); christo.tzachev@gmail.com (C.T.); ohtdv@chem.uni-sofia.bg (D.C.); ohmk@chem.uni-sofia.bg (M.K.); silieva@chem.uni-sofia.bg (S.I.); 4 Lead Biotherapeutics Ltd., 24 Shipka Str., 1504 Sofia, Bulgaria; 5 Department of Chemical Engineering and Analytical Chemistry, University of Barcelona, Martí i Franqués 1-11, E-08028 Barcelona, Spain; raimon_gargallo@ub.edu; 6 Faculty of Physics, Sofia University “St. Kliment Ohridski”, 5 James Bourchier Blvd., 1164 Sofia, Bulgaria; 7 Institute of Molecular Biology ‘‘Roumen Tsanev,’’ Bulgarian Academy of Sciences, ‘‘Acad. George Bonchev’’ Str. 21, 1113 Sofia, Bulgariatedy0890@abv.bg (T.D.-D.);; 8 Department of Oral and Maxillofacial Surgery, Medical University Sofia, 1 “G. Sofijski” Str., 1431 Sofia, Bulgaria; z.mihailova@fdm.mu-sofia.bg; 9 Laboratory of Functional and Nanostructured Polymers, Institute of Polymers, Bulgarian Academy of Sciences, Akad. G. Bonchev Str., Bl. 103A, 1113 Sofia, Bulgaria

**Keywords:** monomethine, cyanine, dye, solid, lipid, nanoparticles, DFT, TDDFT, CD

## Abstract

The development of fluorescence-based methods for bioassays and medical diagnostics requires the design and synthesis of specific markers to target biological microobjects. However, biomolecular recognition in real cellular systems is not always as selective as desired. A new concept for creating fluorescent biomolecular probes, utilizing a fluorogenic dye and biodegradable, biocompatible nanomaterials, is demonstrated. The synthesis of a new dicationic asymmetric monomethine cyanine dye with benzo[d]thiazolium-N-propionamide and chloroquinoline end groups is presented. The photophysical properties of the newly synthesized dye were examined through the combined application of spectroscopic and theoretical methods. The applicability of the dye as a fluorogenic nucleic acid probe was proven by UV-VIS spectroscopy and fluorescence titration. The dye–nucleic acid interaction mode was investigated by UV-Vis and CD spectroscopy. The newly synthesized dicationic dye, like other similar fluorogenic structures, limited permeability, which restricts its use as a probe for RNA and DNA. To enhance cellular delivery, we utilized a patented technology that employs solid, insoluble lipid nanoparticles. This method ensures the complete introduction of the dye into cells while minimizing activity outside the cells. In our study involving two human cell lines, we observed improved penetration through the cell membrane and distinctive selectivity in visualizing nucleic acids within the cytoplasm and nucleus.

## 1. Introduction

In the last two decades, technologies for fluorescence-based detection of biological microobjects have advanced rapidly. The creation of modern confocal fluorescence microscopes makes it possible to observe cells and cell organelles with an excellent resolution. To obtain better-quality images, as well as track some parameters in cells, it is necessary to create of a set of fluorogenic biomarker compounds that will help visualize bio-objects in their natural environment. Additionally, tracking the activity and mechanism of action of new therapeutics is now largely associated with the use of labeling substances, such as fluorescent or fluorogenic biomolecular probes [1]. The term fluorogenic refers to chemical compounds or chemical composites that become fluorescent only after interaction with a specific target. Due to their low toxicity, easy handling and functionalization, cyanine dyes are useful tools for rationalizing the mechanism of bioactivity of new drugs in the search for new pharmacophores [2]. Even small changes in the cyanine dyes’ structure lead to significant changes in their photo-physical properties [3,4,5,6,7,8,9,10]. Generally, in molecular solutions, cyanine dyes do not demonstrate intrinsic fluorescence. Upon binding to a biomatrix the chemical structures of cyanines become rigid and the absorbed light energy cannot be released as heat during the relaxation, which causes a significant increase in the fluorescence after DNA, RNA, or protein binding [2,3,4,5,6]. The structural changes are function-determining, and the different substituents on the cyanine aromatic units influence the polynucleotide binding mode and affinity, spectroscopic response, and biological activity [9,10]. In this way, attaching various functionalities to the same molecule brings value to cyanine dyes for selective binding and labeling different bio-objects [4,5,6,7,8,9]. For example, a series of new asymmetric monomethine cyanine dyes, thiazole orange (TO) and thiazole orange homodimer (TOTO), with hydroxypropyl functionality showed selectivity towards GC-DNA base pairs over AT-DNA, which included both binding affinity and a strong fluorescence response [8]. The dyes described in the same work also inhibited tumor cell growth in micromolar concentrations, efficiently entering cells and emitting a strong green fluorescence, when accumulated in cytoplasm organelles [8]. Thus, novel dyes combine highly selective fluorescent intracellular probing with strong antiproliferative activity [8]. In this regard, researchers found that bulky substituents attached to the quinolone part affected minor groove binding instead of intercalation [9], while bulky phosphonium substituents are able to differentiate between homo and alternating AT-DNA sequences by forming fluorescent dimers within the DNA minor groove [6]. Considerable efforts have been made to develop small-molecule-based fluorescent dyes for RNA imaging in live cells [11,12,13,14,15,16]. The weak nuclear membrane permeability of the DNA and the RNA-specific dyes is also a big disadvantage [17]. In general, the molecular design of new target-specific fluorescent compounds is key to addressing these problems. The most successful and promising was the study of Lu and coworkers [18], who described a new RNA-selective fluorescent dye integrated with thiazole orange and a styryl moiety. The dye shows a better nucleolus-RNA staining and imaging performance in live cells than commercial stains like SYTO RNASelect. Regardless of the prominent advantages of cyanine dyes, the application in fluorescence diagnostics of some representatives from this class of compounds is accompanied by a number of difficulties. When making some changes in the chemical structure of the cyanine dyes, related to specific applications, it is possible to produce undesirable properties that complicate the further practical application. For example, when aiming to increase the affinity of dyes to certain hydrophobic regions in target bioobjects, unwanted aggregation may occur. In addition, highly hydrophobic dyes can cause unwanted precipitation of a given biopolymer after interacting with it. The application of some cyanine dyes is hindered by their low water solubility, low penetration through cell membranes, and low chemical and photostability under specific application conditions. Very often, the desire to enhance the affinity of dyes to specific regions in nucleic acids (NAs) by introducing bulky substituents results in difficult penetration through the cell membrane and through the nuclear membranes of living cells. Then, interventions such as electroporation are required. Such interventions inevitably move the current state of the cells away from normal and discredit the experiments. To overcome all mentioned problems, one can use different nanocarriers loaded with the target dyes. The masking effect or so called “stealth” effect of nanocarriers can significantly improve cellular membrane penetration, and in the best-case scenario, upon the intracellular degradation of the nanoparticles, can increase the biotargeting of the in-cell-released fluorescent probes.

The synthesis and photophysical properties of novel dicationic asymmetric monomethine cyanine dye with benzo[d]thiazolium-N-propionamide and chloroquinolinium end groups are described in the present article. The nucleic acid differentiation and preference were studied as well.

The newly synthesized dye, characterized as a dicationic molecule, exhibits significantly limited permeability to cellular membranes. This trait is common among similar fluorogenic dyes, and it poses challenges for their use as effective probes for RNA and DNA detection. To address this limitation and facilitate a more efficient cellular uptake, we implemented a patented intracellular delivery technology, CellInject, which employs solid, insoluble lipid nanoparticles and is designed as a transport tool for such “difficult” molecules.

CellInject is the first particulate system designed to transport active substances into target cells, where the release is exclusively initiated following intracellular activation, ensuring a lack of release in aqueous media prior to internalization. The targeted release mechanism operates independently of the drug concentration gradient and is propelled by the particles’ strong affinity for cellular membranes.

To assess the effectiveness of our delivery method, we conducted a series of experiments to evaluate cellular uptake in two distinct human cell lines: HeLa Kyoto cells and human mesenchymal stem cells derived from the apical papilla (SCAP) of adult teeth with incomplete root development. Both human mesenchymal stem cells and HeLa immortalized cancer cells are well-established model systems with high proliferation rates, making them suitable for investigating various cellular processes. Our objective is to employ this innovative technology as a fluorogenic probe for cell staining and visualization of nucleic acids.

## 2. Results and Discussion

### 2.1. Synthesis

**Scheme 1:**
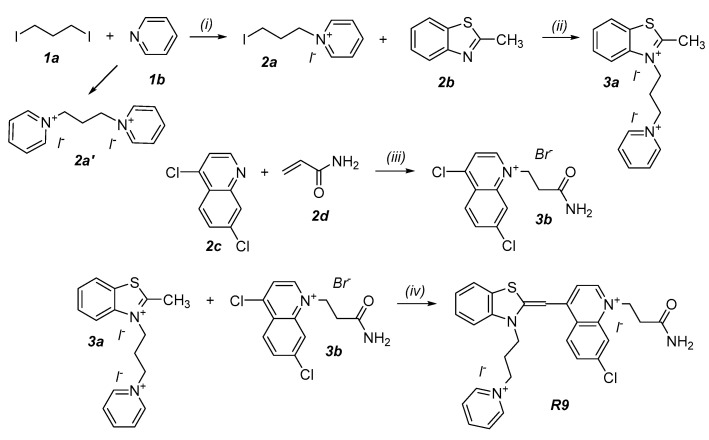
Reactants, conditions, yields: (**i**) **1a**, **1b**, acetone, r.t., two weeks in a dark, 91%; (**ii**) **2a**, **2b**, NMP, 145 °C, 3h, Et_2_O, 74%; (**iii**) **2c**, **2d**, AcOH, 48% HBr, 68%; (**iv**) **3a**, **3b**, DIPEA, EtOH:DCM = 1:1, 71%.

We performed a quaternization reaction between 1,3-diiodopropane (**1a**) and pyridine (**1b**) in acetone as a solvent at room temperature by a previously described synthetic strategy [9]. The quaternary product 1-(3-iodopropyl)pyridinium iodide (**2a**) and the bis-quaternary by product 1,1′-(1,3-propanediyl)bis(pyridin-1-ium) diiodide (**2a’**) (Scheme 1) are not soluble in diethyl ether. Therefore, we isolated them from the reaction mixture via precipitation with diethyl ether. In this way, the soluble component in ether-unreacted starting materials **1a** and **1b** (Scheme 1) remains in the solution. The target compound **2a** was separated from the side product **2a’** by short-path silica flash chromatography with eluent DCM: MeOH, with increasing polarity. The polar dicationic by product **2a’** is retained on the silica and cannot be eluted even with a solvent mixture with significant polarity. In this way, the outgoing solvent contains only the pure target compound 2a. The quaternization reaction of **2a** and 2-methylbenzothiazole (**2b**) performed in N-methyl-2-pyrrolidone (NMP) as a solvent [9] produced 2-methyl-3-(3-(pyridin-1-ium-1-yl)propyl)benzo[d]thiazol-3-ium diiodide (**3a**) (Scheme 1).

The Michael’s type 1,4-addition reaction of 4,7-dichloroquinoline (**2c**) to acrylamide (**2d**) in acetic acid solution produced the intermediate 1-(3-amino-3-oxopropyl)-4,7-dichloroquinolin-1-ium bromide (**3b**) [13] in a very good yield (Scheme 1). The quaternary salt **3b** is moisture-sensitive and unstable; therefore, it was used directly in the next reaction step without further purification.

The condensation reaction between heterocyclic N-quaternary onium salts **3a** and **3b** (Scheme 1) was performed at room temperature in the presence of base N-ethyldiisopropyl amine (DIPEA) in a mixture of ethanol–dichloromethane 1:1 mL as a solvent. Before the addition of DIPEA, two solid phases of the starting quaternary salts **3a** and **3b** were present. The reaction occurs from suspension. As the reaction progresses, three solid phases are observed in the reaction volume in addition to the liquid phase: two of the starting reactants and one of the products. After the end of the synthesis (TLC monitoring), the red-violet precipitate formed was the only phase left in the reaction volume. It was filtered, washed with ethanol and recrystallized from a mixture of ethanol: dichloromethane (1:1). After filtration of the hot solution, the dichloromethane was removed under reduced pressure. The evaporation of the dichloromethane and subsequent cooling of the solution resulted in a needle-like pale violet precipitate, which was filtered and dried in a desiccator. The purity of the target dye (E)-1-(3-amino-3-oxopropyl)-4-((3-(3-(pyridin-1-ium-1-yl)propyl)benzo[d]thiazol-2(3H)-ylidene)methyl)quinolin-1-ium diodide (**R9**) and its chemical structure were prove, by NMR spectroscopy, mass spectrometry, UV-VIS, and fluorescence spectroscopy.

### 2.2. Quantum Chemical Computations

Theoretical calculations were carried out using Gaussian 16A.03 software for quantum chemical computations [19]. Geometric optimization of the new dicationic dye **R9** in the gas phase and water solvent was performed using the DFT method at the B3LYP/6-31+G(d,p) level. Vibrational and electronic spectral characteristics were calculated based on the optimized geometry of **R9**. Theoretical parameters reflecting the electronic density distribution in the dye molecule were also calculated: atomic charges and electrostatic potential at the nuclei (EPN). Atomic charges were calculated according to three different population analysis schemes: Hirshfeld, MK, and NPA. Changes in the electron density distribution between the ground and the first excited electronic state of the dye’s molecule were analyzed. UV-VIS and fluorescence spectra of the dye were modeled based on the optimized geometry.

Calculating UV-VIS spectra of cyanine dyes is challenging because most TDDFT functionals greatly overestimate the energy of their electronic transitions. In our previous study, the applicability and accuracy of several DFT functionals for calculating the absorption and fluorescence maxima of monomethine cyanine dyes were assessed [20]. It was concluded that the pure exchange functionals (M06L, HFS, HFB, B97D) show the best performance for the theoretical evaluation of the absorption and fluorescence characteristics of cyanine dyes. The dye absorption maxima computed with HFB and HFS functionals appeared to agree very well with the experimental values [20]. Two functionals were applied in the present study, PBE0 and HFS, to predict the photophysical properties of the dye **R9**. HFS was chosen based on our benchmark studies and PBE0 for comparison, as this is one of the most applied functionals for excited state studies. Using the TDDFT approach, the absorption transition energies and oscillator strengths (f) were calculated (Table 1). The obtained theoretical results were validated against the absorption experimental data. It can be seen from the data in Table 1 that the TDHFS/6-31+G(d,p) calculations reproduced the experiment better.

**Scheme 2:**
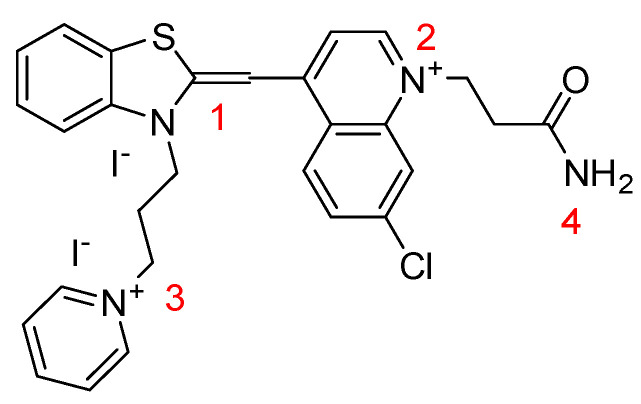
Numbering of the nitrogen atoms in the **R9** molecule.

The computed electronic parameters for the **R9** molecule—NBO, MK, and Hirschfeld atomic charges, as well as the electrostatic potential at the nuclei (EPN)—are summarized in Table 2. The numbering of the nitrogen atoms corresponds to Scheme 2. Galabov and coauthors [21] have shown that Hirshfeld charges perform better in describing the properties of aromatic molecules. The data in Table 2 confirm this conclusion, showing that three of the nitrogen atoms (N1, N2, N3) are positively charged as expected. The nitrogen atoms N2 and N3 have a greater positive charge, and N4 is negatively charged. EPN values accurately reflect the change in electron density without additional approximations that are intrinsic to atomic charges depending on their definition. The more negative the EPN value, the more negatively charged the atom. The trend in the calculated EPN values corresponds to Hirshfeld charges.

**Figure 1:**
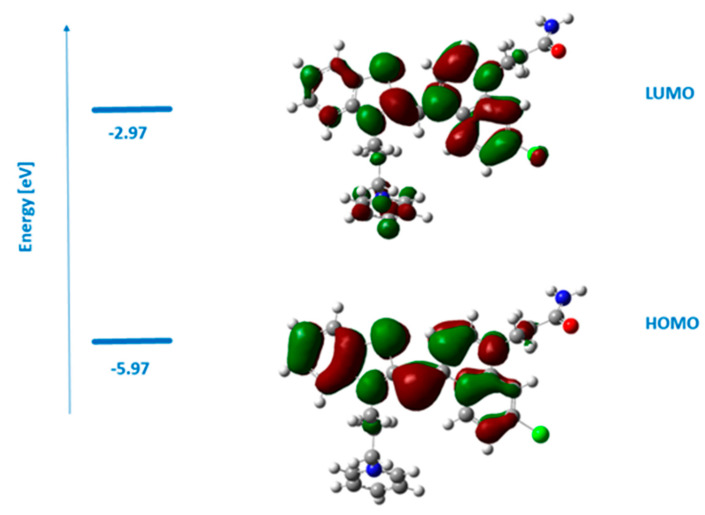
HOMO and LUMO shape representation for the ground state of dye **R9** from B3LYP/6-31+G(d,p) computations in water medium.

The electron density distribution in the ground and excited states was ascertained with molecular orbital shape analysis. Figure 1 shows the shapes of the HOMO and LUMO orbitals from the B3LYP/6-31+G(d,p) calculations in water medium. The HOMO and LUMO orbitals are delocalized on the benzothiazole and quinoline rings. The density in the HOMO orbital is more evenly distributed, while the electron density in the LUMO orbital is drawn towards the quinoline heterocycle, which corresponds to an intramolecular charge-transfer (ICT) electronic transition.

DFT calculations reveal that the largest positive charge is localized at the nitrogen atom N3 in the pyridinium moiety of the side group attached to the benzothiazole. The presence of this positively charged and bulky functionality would hinder intercalation and facilitate groove-binding interaction between the dye and the nucleic acid.

### 2.3. Photophysical Properties

**Figure 2:**
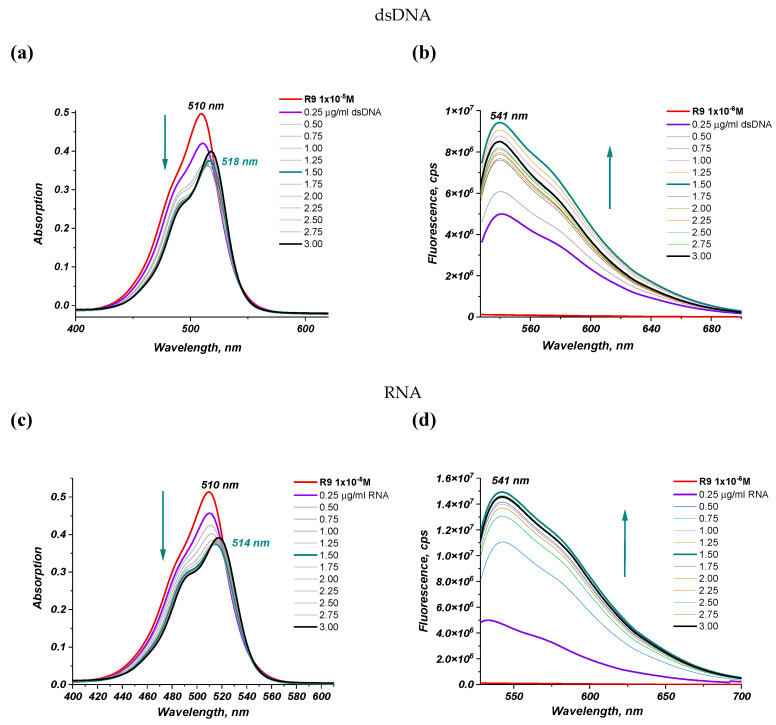
UV-VIS and fluorescence properties of dye **R9** and its complexes with dsDNA (**a**,**b**) and RNA (**c**,**d**)**.** The arrows represent the band intensity change upon an increasing NA concentration.

The main photophysical properties of the new asymmetric dicationic monomethine cyanine dye **R9** were characterized using UV/Vis and fluorescence spectroscopy. Figure 2 presents the absorption and fluorescence spectra obtained by titrating a dilute TE buffer solution of the dye with dsDNA and RNA. The absorption and fluorescence maxima of dye **R9**, both in the absence and presence of dsDNA and RNA, are summarized in Table 3.

The absorption spectrum of the pure dye **R9** at a concentration of 10 μM in a buffer solution (red line, Figure 2a,c) shows an intensive sharp peak at 510 nm (molar absorptivity: 50,540 L.mol^−1^.cm^−1^), which is characteristic of monomeric asymmetric monomethine cyanine dyes (Table 3). The formed **R9-DNA** complex absorbs at 518 nm with a lower molar absorptivity of about 37,700 L.mol^−1^.cm^−1^, demonstrating a bathochromic and pronounced hypochromic shift. The **R9-RNA** complex absorbs at 514 nm (molar absorptivity at 37,210 L.mol^−1^.cm^−1^), showing the same trend—bathochromic shift and pronounced hypochromic effect.

As the concentration of NA increases, the system reaches saturation (indicated by the green line in Figure 2a,c). Once the lowest intensity of the absorption band is achieved at saturation, further addition of NA results in an increase in band intensity (shown by the black line in Figure 2a,c), with the effect being much more pronounced for DNA.

The non-covalent binding of small molecules to DNA involves intercalation between base pairs, groove binding, and/or external electrostatic interaction. When small molecules intercalate into DNA, the absorption spectrum of the molecule shows a bathochromic and hypochromic shift [22,23,24]. The bathochromic effect is the result of a decrease in the π/π* transition energy due to the coupling of the π bonding orbital of the nucleic acid base pairs with the empty π* antibonding orbital of the ligands. When intercalation occurs, typical values of hypochromicity (higher than 35%) and of bathochromicity (red shift, ∆λ > 15 nm) in the visible band are expected. These are typical values determined for long duplex DNA where end stacking is not significant, like those studied here [25,26].

In the case of groove-binding interactions, a smaller or no bathochromic shift is observed. Due to the less direct contact between π-systems, changes in the absorption spectra are less remarkable for groove binding or outside binding. In these cases, typical red shifts of under 8 nm have been described [27,28]. External electrostatic interactions result in a hyperchromic effect [29].

In our case, the bathochromic and hypochromic shifts observed in the absorption bands of the dye as dsDNA and the RNA concentration increase point to a groove-binding mechanism [25,26]. The specific hyperchromic effect observed after saturation can be attributed to additional electrostatic binding of the dye to the nucleic acid, with this effect being more pronounced for dsDNA. This behavior is well-explained when considering the dicationic nature of the **R9** dye.

Dye **R9** exhibits extremely low intrinsic fluorescence in an aqueous buffer solution at pH = 8 (red line, Figure 2b,d). The **R9-DNA** complex fluoresces at 541 nm. The significant increase in fluorescence intensity with an increasing nucleic acid (NA) concentration clearly indicates a strong interaction between the **R9** dye and the NA. The dye shows greater sensitivity to RNA, with a 136-fold increase in fluorescence intensity (Table 3).

### 2.4. Circular Dichroism

Circular dichroism (CD) spectroscopy is an analytical technique that may help to define the binding mechanism of the **R9** dye to dsDNA and RNA. This technique is sensitive to conformational changes of polynucleotides induced by the complexation with small molecules.

The CD spectrum of B-DNA is characterized by negative and positive signals around 250 and 275 nm, whereas the CD spectrum of A-RNA shows only a positive signal around 275 nm. Upon addition of the **R9** dye, a small reduction in the intensity of these signals, both for dsDNA and RNA, was observed. A detectable decrease in the polynucleotide CD spectrum is normally a sign of changes in helical chirality due to the intercalation of small molecules or binding of bulky compounds within the polynucleotide groove [30].

**Figure 3:**
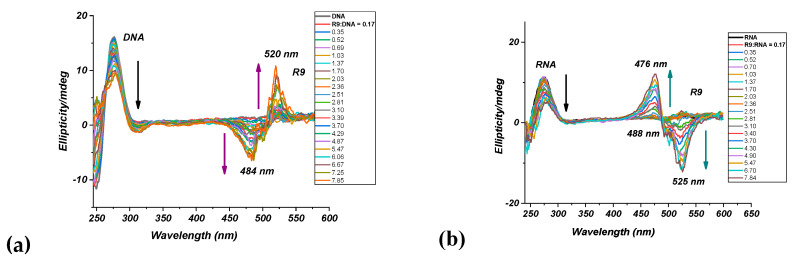
ICD in a solution of (**a**) dsDNA and (**b**) RNA with an increasing **R9** dye concentration. The arrows represent the band intensity change upon an increasing NA concentration.

The **R9** dye molecule is achiral and does not exhibit an intrinsic CD spectrum. The binding of a small and achiral molecule to a chiral polynucleotide could produce the appearance of an induced CD (ICD) signal in the CD spectrum of the dye. The ICD signal can be useful to characterize the mode of interaction (intercalation, groove binding, dye stacking, etc.) [8]. Figure 3a shows that the addition of the **R9** dye to the dsDNA produced the appearance of an ICD signal characterized by negative and positive signals at 484 and 520 nm, respectively, of similar intensity. This spectral signature is known as an induced exciton CD signal, showing a characteristic bisignate shape with one positive and one negative band located on either side of the absorption maximum of the free ligand [31]. An induced exciton CD signal is indicative of groove binding or an external stacking-binding mode. Induced exciton CD signals are typically not observed for intercalators, which are usually denoted by weak negative ICD bands. The biphasic shape of the ICD signal observed in both cases in Figure 3 is most likely due to exciton coupling of stacked **R9** molecules on the grooves, as pointed out from the spectral shifts shown in Figure 2. Interestingly, for the interaction of the **R9** dye with dsDNA, the negative ICD band at a lower wavelength and the positive ICD band at a higher wavelength are characteristic of chromophores that have a right-handed orientation, as expected for dyes bound to B-form DNA [32]. Conversely, the opposite orientation of the chromophore would be expected for the interaction of **R9** with RNA. It is known that the B-DNA helix shows different spatial features than the RNA helix, like the number of bases or the pitch, among others. As a result, the major groove of RNA is narrower and deeper than in B-DNA, whereas the minor groove is wider and shallower than in B-DNA. These features would affect the binding of **R9**, producing a different docking in both cases.

### 2.5. Cellular Dye Uptake and Distribution of **R9** when Loaded on CellInject

#### 2.5.1. Cellular Toxicity of CellInject Placebo and Dye-Loaded Particles

**Figure 4:**
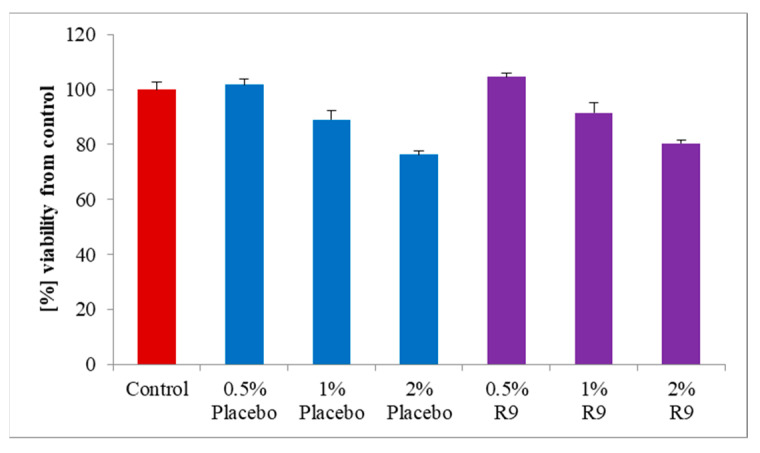
Viability of HaCaT cells treated for 30 min with placebo and **R9**-loaded CellInject, which contained lipid fractions of 0.5%, 1%, and 2% and dye concentration of 30 nM (mean ± SD, n = 3).

We conducted toxicity tests on the human HaCaT keratinocyte cell line (refer to Figure 4). Keratinocytes, which are epidermal-derived epithelial cells, were chosen due to the potential application of the dye-loaded particles on human skin and other epithelial tissues for diagnostic purposes. In our study, we evaluated 0.5%, 1%, and 2% dispersions of a placebo, alongside equivalent dispersions containing **R9** at a concentration of 30 nM, administered for a duration of 30 min. The results indicated no statistically significant differences among the samples. Furthermore, the tested formulations did not exhibit any noticeable toxic effects on the viability of HaCaT cells. These findings suggest that CellInject possesses minimal toxicity and may thus be deemed safe for relevant applications. We note that for the visualization tests, CellInject particles are diluted in the medium down to concentrations below 0.1% *w*/*w*.

#### 2.5.2. HeLa Kyoto Cell Line

**Figure 5:**
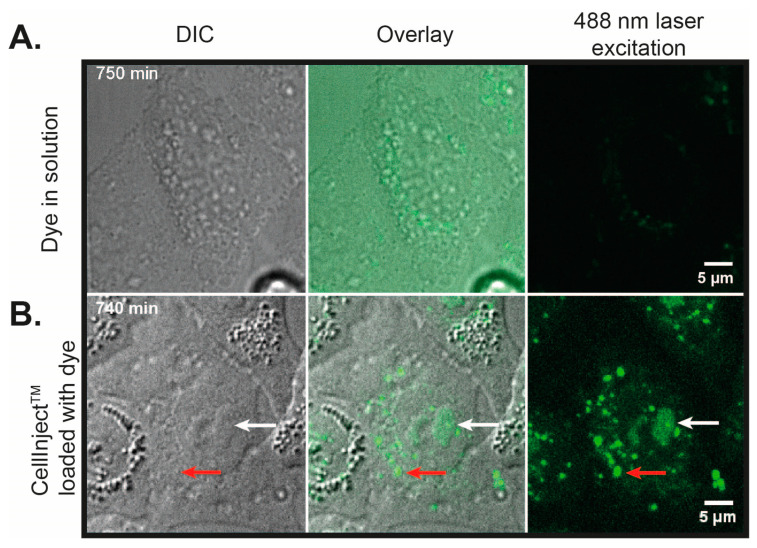
Fate of CellInject lipid particle system, loaded with fluorescence dye, 30 nM, on *HeLa Kyoto* cells. (**A**) HeLa Kyoto cells imaged at 750 min after treatment with dye solubilizate. Images from the DIC channel (left), 488 nm channel (right), and overlay of both channels (center) are shown. No dye internalization and no fluorescence are observed inside the HeLa cell. (**B**) The dye is integrated within CellInject particles. Massive fluorescence due to dye–RNA complexes is observed in the cytoplasm and nucleoli of the HeLa Kyoto cells (indicated with red and white arrows, respectively). DIC (left), overlay (center), and green fluorescence (488 nm laser) images.

Different cell types have different rates of cellular uptake, which necessitates testing the same molecular probes on different cell lines in order to find cell- and organelle-selective biomolecular probes. To study **R9** dye uptake, we treated HeLa Kyoto human cervical cancer cells with the dye and acquired images in two channels. Images in the first channel were acquired via differential interference contrast (DIC), which allowed for imaging of nonlabeled cells. In the second channel, cells were excited with a 488 nm laser, and fluorescence emission was acquired in the range between 500 and 550 nm. Treatment of the cells with **R9** dye solution (Figure 5A) showed a very low intrinsic emission, with no emission spots detected within the examined cells at the given concentration. The absence of emission is illustrative of the inability of the dye itself to enter HeLa cells. Conversely, the loaded CellInject (Figure 5B) exhibited no dye release into the culture medium, correlating with the absence of fluorescence in said medium. However, strong and sustained fluorescence was observed after 60 min of the treatment within cells until the end of the experiment (13 h), attributed to enzymatic degradation of the particles by intracellular enzymes. Pronounced dye accumulation was noted in the cytoplasm and the nucleoli (indicated by red and white arrows, respectively).

In the parameters set in these conditions, there is no fluorescence outside the cells. The fluorescence occurs as a result of CellInject particles’ degradation by intracellular enzymes with release of the free dye, which further forms fluorescent complexes with intracellular RNA, as is indicated by preferential binding of the nucleoli. The lipid matrix of CellInject as an SLN-based system can protect labile active ingredients from hydrolysis and/or oxidation [17]. The control with the pure particles shows no fluorescence within HeLa Kyoto cells (Figure 5A). Conversely, the loaded CellInject identified in Figure 5B exhibits no dye release into the culture medium, correlating with the absence of fluorescence in said medium. However, robust fluorescence becomes apparent within cells after 30 min, attributed to enzymatic degradation of the particles. Videos of the experiment can be found in the ESI.

#### 2.5.3. Human Mesenchymal Stem Cells (MSCs)

Mesenchymal stem cells are undifferentiated fibroblast-like cells that express specific molecular markers. They can differentiate into multiple cell types and are capable of self-renewal [33]. Therefore, these cells are useful platform for studying cellular processes and testing various new methods and techniques [34]. Some of the most easily accessible MSC are those of dental origin [35]. Routinely extracted teeth are usually considered biological waste without paying attention to the properties of the non-differentiated cells they contain. Dental MSCs can differentiate into odontoblasts, cementoblasts, osteoblasts, chondroblasts, myocytes, epithelial cells, nerve cells, hepatocytes, adipocytes, etc. The specific differentiation is highly dependent on the local environmental factors, i.e., pH, growth factors, active molecules, and signals [36,37,38]. Huang et al. [39] isolated growth organ cells of underdeveloped teeth and demonstrated their ability to differentiate odontogenically when implanted in mice. They also proposed a method of using these cells in the re-implantation and transplantation of teeth. The ability of these cells to build underdeveloped dental tissues, according to the authors, suggests great possibilities for their application in dentistry for regeneration of the tooth root, pulp, and periodontium.

We initially used stem cells from the apical papilla (SCAP). SCAP are suitable for in vitro research with the intention of their application in regenerative medicine and bioengineering due to their rapid multiplication, easy cultivation, and ability to remain undifferentiated during division. Due to the tremendous interest to the SCAP cells in tissue and organ regeneration and engineering, we decided to test the ability of dye **R9** to play the role of a specific fluorescent binder for cellular organelles or SCAP’s cellular compartments. We investigated the cellular uptake and the fluorescent binding abilities of **R9** in SCAP cellular culture.

**Figure 6:**
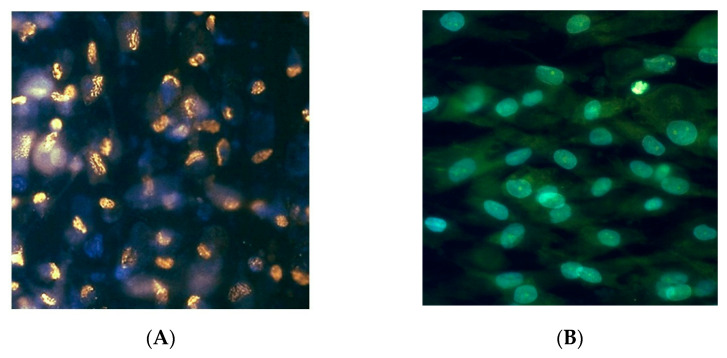
(**A**) Living SCAP cells stained with 2 μM **R9**, where the dye stayed outside the nuclei; (**B**) nucleoli-like structures in the formalin-fixed cells are stained. Magnification ×60.

After adding dye **R9** to SCAP cells, we examined the penetration through the cellular membrane and the distribution of the dye in the cell. We found that the 2 μM concentration seemed not to be sufficient for nuclear membrane penetration, as the dye remained in the cytoplasm and stained cytoplasmic structures located closely to the nuclei. The granular structure of the stained organelles resembles the granulated endoplasmic reticulum (Figure 6A). When the cells were formalin-fixed, the 2 μM dye penetrated the cell nuclei and concentrated in structures resembling the nucleoli (Figure 6B).

**Figure 7:**
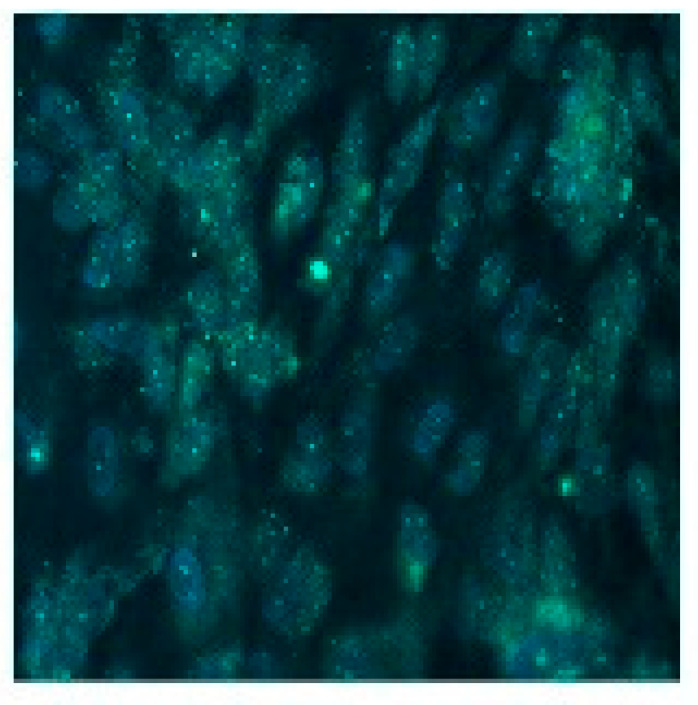
SCAP stained with **R9** dye nanoparticle solution. Cytoplasmic ER-like structures and nuclear nucleoli-like structures concentrate the dye (green). Counterstain: DAPI blue. Magnification ×60.

CellInject particles, which were loaded with **R9** dye, were administered to living SCAP cells at a concentration of 30 nM. Following a three-hour incubation period, the dye was detected in both the cytoplasm and the nucleus. Within the cytoplasm, the staining displayed a granular structure akin to that of the endoplasmic reticulum (ER). The nucleus exhibited minimal staining; however, distinct fluorescence signals were concentrated in regions that resembled nucleoli (Figure 7).

**Figure 8:**
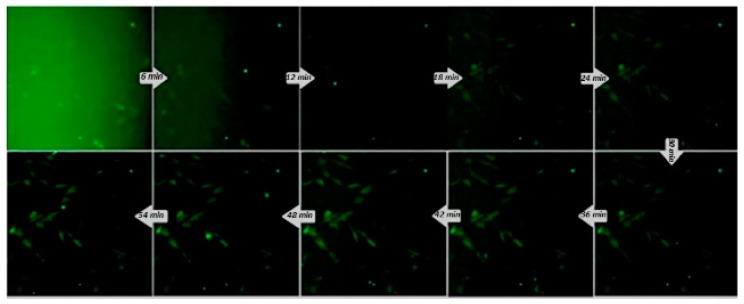
SCAP stained by **R9** dye nanoparticle solution (green). Magnification 20×. Note: Images are taken from a dynamic file, which accounts for the low resolution.

To check the stealth effect on the cells of the newly synthesized dye encapsulated within CellInject, we observed the samples for 1 h by photographing them at intervals of 6 min (Figure 8).

The data presented clearly demonstrate a notable fluorescent signal emerging after a definite time needed for the particles to penetrate the cells and to deliver the fluorogenic dye. Neither the CellInject alone nor those loaded with the dye have significant absorption or emission within the tested wavelength range (400–600 nm). In contrast, the dye alone exhibits robust and detectable emission when it binds to RNA located in the cytoplasm and nuclei of the cells, indicating an intracellular release of the cationic dye.

The CellInject particles possess a distinctive characteristic in that they do not release the loaded active substances in aqueous media, as evidenced by the lack of detectable fluorescence observed outside the cells during the experiment. The predominant mechanism underlying dye release is cellular internalization, whereby the particles traverse the cell membrane into the cytoplasm. Subsequent degradation of the nanoparticles facilitates the dye release, allowing it to bind to RNA in the cytoplasm. The resultant fluorescent signal is sufficiently intense to enable visualization and monitoring of nucleic acids and the biological processes in which they are engaged in living cells.

This innovative delivery system is designed to encapsulate the dye, ensuring its effective introduction into the cytoplasm while simultaneously minimizing any potential interactions or side effects in the extracellular environment. By using lipid nanoparticles, we leverage their natural ability to merge with cellular membranes, promoting enhanced internalization of the dye.

Our findings revealed that this approach significantly improved the dye’s penetration through cell membranes compared to traditional methods. Moreover, we noted an increased selectivity in visualizing nucleic acids located within the cytoplasm and the nucleus, enabling more precise imaging outcomes. This advancement not only broadens the potential applications of the dye but also enhances its utility in molecular biology and diagnostic research.

## 3. Experimental Part

### 3.1. General

All solvents used in the present work are HPLC grade and commercially available. The starting materials **1a**, **1b, 2b, 2c**, and **2d** are commercially available and they were used as supplied. Intermediates **3a** and **3b** were prepared by previously described reaction procedures [9]. **R9** is slightly soluble in redistilled water and DMSO; therefore, fresh stock solution (1 mM) was prepared and further diluted with TE buffer (Tris-HCl 10 mM pH 8.0; EDTA 1 mM, pH 8.0).

NMR spectra (^1^H-, ^13^C-NMR) were obtained on a Bruker Avance II + NMR spectrometer operating at 500 MHz for ^1^H- and 125 MHz for ^13^C-NMR in DMSO-d_6_ as a solvent. The chemical shifts are given in ppm (δ) using tetramethylsilane (TMS) as an internal standard.

UV-VIS spectra were measured on a Unicam 530 UV-VIS spectrophotometer (ThermoFisher Scientific Co., Waltham, MA, USA) and the fluorescence spectra were obtained on a Fluorolog 2 fluorescence spectrophotometer (Horiba, Ltd., Kyoto, Japan). The absorption and emission properties of the **R9** dye were investigated in TE buffer with pH = 8 in the absence and presence of dsDNA and RNA. The fluorescence spectrum of the **R9** dye was recorded with a FluoroMax-2 spectrofluorometer. All UV-VIS and fluorescence titrations were carried out by maintaining the **R9** dye concentration constant while varying the DNA and RNA concentrations. After the addition of the aliquots of NA (5 μL 0.25 μg/mL NA solution), the solution was equilibrated for 5 min before recording the spectrum. The width of both slits was Δλ = λ_central_ ± 5 nm (FWHM). In all measurements, the excitation wavelength was 5 nm before the absorption maximum. Fluorescence detection started 20 nm after the excitation wavelength.

The progress of the chemical reactions was followed using thin-layer chromatography (TLC) using ALUGRAM^®®^ SIL G/UV 254-60 (Macherey-Nagel GmbH & Co. KG, Düren, Nordrhein-Westfalen, Germany) ready-to-use plates, with the thickness of the silica layer at 0.2 mm. Liquid chromatography–mass spectrometry (LC-MS) analyses were carried out on a Q Exactive^®®^ hybrid quadrupole-Orbitrap^®®^ mass spectrometer (ThermoFisher Scientific Co., Waltham, MA, USA) equipped with an HESI^®®^ (heated electrospray ionization) module, TurboFlow^®®^ Ultra High Performance Liquid Chromatography (UHPLC) system (ThermoFisher Scientific Co., Waltham, MA, USA) and HTC PAL^®®^ autosampler (CTC Analytics, Zwingen, Switzerland). Chromatographic conditions: The chromatographic separations of the analyzed compounds were achieved on a Nucleodur C18 Isis (150 × 2.1 mm, 3.5 μm) analytical column (Macherey-Nagel GmbH & Co. KG, Düren, Nordrhein-Westfalen, Germany) using gradient elution at a 300 L/min flow rate. The used eluents were A—0.1% formic acid in water; B—0.1% formic acid in ACN.

Mass spectrometry conditions: Fullscan mass spectra over the *m*/*z* range 100–1500 were acquired in the positive ion mode at resolution settings of 70,000. The mass spectrometer operating parameters used in the positive ionization mode were as follows: spray voltage −4.0 kV; capillary temperature −320 °C; probe heater temperature −300 °C; sheath gas flow rate 27 units; auxiliary gas flow 7 units; sweep gas 2 units (units refer to arbitrary values set by the Q Exactive Tune Q Exactive HF Tune 2.4 software); and S-Lens RF level of 50. Nitrogen was used for sample nebulization and collision gas in the HCD cell. The main ions were [M + H]^+^. Data acquisition and processing were carried out with the XCalibur^®®^ ver 2.4 software package (ThermoScientifc Co, USA).

Placebo CellInject nanoparticles were obtained according to a described procedure (WO2019116062 and US12036329 (B2) [40,41]). Briefly, the lipid blend was mixed with the surfactant and water and subsequently subjected to a series of controlled temperature changes utilizing the phase-inversion method. The particles were loaded with dye at a concentration of 30 nM. The size of the nanoparticles was 35–55 nm.

HeLa Kyoto cells were cultured in Dulbecco’s Modified Eagle’s Medium (DMEM) from Thermo Fisher Scientific supplemented with 10% fetal bovine serum (FBS) (ThermoFisher Scientific Co., Waltham, MA, USA) and 100 units/mL penicillin (ThermoFisher Scientific Co., Waltham, MA, USA) and 100 μg/mL streptomycin (ThermoFisher Scientific Co., Waltham, MA, USA) at 37 °C and 5% CO_2_. For micro-irradiation and image acquisition, cells were plated in MatTek glass-bottomed dishes (MatTek, Ashland, MA, USA) (~40% confluence), then cultured for 24h, washed with PBS, and supplemented with 2 mL FluoroBrite DMEM medium (ThermoFisher Scientific Co., Waltham, MA, USA) containing 10% FBS and 2 mM GlutaMAX (ThermoFisher Scientific Co., Waltham, MA, USA). Before imaging, 100 ul CellInject particles was added. Image acquisition was performed on an Andor Revolution system using a Nikon 60× (NA1.2) water immersion objective and iXon897 EMCCD camera. Images were acquired in two channels, using 488 nm laser excitation in 7 Z planes, with 0.5 μm plane spacing and a single plane in bright-field DIC. The resolution of the entire chip of the camera used is 512 × 512 pixels. The individual videos and pictures were cropped to 191 × 206 pixels. One pixel is about 200 nm.

### 3.2. Synthesis of 1-(3-Amino-3-oxopropyl)-7-chloro-4-((3-(3-(pyridin-1-ium-1-Yl)propyl)benzo[d]thiazol-2(3H)-ylidene)methyl)quinolin-1-ium iodide (**R9**)

We finely ground 0.52 g (1 mmol) of 2-methyl-3-(3-(pyridin-1-ium-1-yl)propyl)benzo[d]thiazol-3-ium diiodide (**3a**) and 0.42 g (1.2 mmol) of 1-(3-amino-3-oxopropyl)-4,7-dichloroquinolin-1-ium bromide (**3b**) in a mortar and transferred that to a 50 mL round-bottomed reaction flask equipped with an electromagnetic stirrer. The flask was closed with a rubber septum and the air was evacuated by a vacuum pump. The flask was flushed with argon, and 5 mL ethanol and 5 mL ethyl acetate were added by syringe. The reaction mixture was stirred intensively at room temperature for 15 min. We added 0.28 g (0.38 mL, 2.2 mmol) DIPEA dropwise to the mixture over about 15 min. After the addition of Huenig’s base, the reaction mixture was stirred vigorously at room temperature for one additional hour. Stirring was stopped and the dichloromethane was evaporated on a rotary vacuum evaporator to a volume of about 5 mL. The mixture was allowed to stand in the refrigerator overnight and was filtered in a Buechner funnel and washed with three 10 mL portions of ethanol. The resulting bright red amorphous precipitate was dried in a vacuum desiccator. Yield of the crude product: 0.54 g (71%). The dye was purified by double recrystallization from ethanol:dichloromethane (1:1), obtaining 1-(3-amino-3-oxopropyl)-7-chloro-4-((3-(3-(pyridin-1-ium-1-yl)propyl)benzo[d]thiazol-2(3H)-ylidene)methyl)quinolin-1-ium iodide (**R9**) as red needles: ^1^H-NMR (300 MHz, DMSO_d6_, δ (ppm)): 2.51–2.58 m (2H, CH_2_); 2.74 t (2H, J = 6 Hz); 4.81–4.95 m (6H, N^+^CH_2_); 6.90 s (1H, CH); 7.01 s (1H, CH); 7.39 d (1H, J = 7 Hz, CH); 7.46 d (1H, J = 7 Hz, CH); 7.49 d (1H, J = 8 Hz, CH); 7.66 dd (appears as t, 1H, J = 8 Hz, J = 8 Hz); 7.80 d (1H, J = 7 Hz, CH); 7.82 d (1H, J = 7 Hz, CH); 7.90 d (1H, J = 8 Hz, CH); 8.10 d (1H, J = 8 Hz, CH); 8.16 dd (appears as t, 1H, J = 7 Hz, J = 7 Hz); 8.23 d (1H, J = 7 Hz, CH); 8.29 s (1H, CH); 8.46 dd (1H, J = 9 Hz, J = 12 Hz); 8.58 dd (t, 1H, J = 7 Hz, J = 7 Hz); 8.64 dd (appears as t, 1H, J = 9 Hz, J = 9 Hz, CH); 8.84 d (1H, J = 9 Hz, CH); 9.14 d (1H, J = 6 Hz, CH). ^13^C-DEPT: 28.73 (CH_2_); 33.93 (CH_2_); 42.93 (CH_2_); 50.68 (CH_2_); 57.86 (CH_2_); 83.30 (CH); 107.96 (CH); 113.19 (CH); 117.44 (CH); 123.08 (CH); 124.90 (CH); 126.89 (CH); 128.06 (CH); 128.15 (CH); 128.30 (CH); 128.42 (CH); 144.78 (CH); 144.84 (CH); 145.63 (CH); 145.68 (CH). HPLC-ESI-MS: Chemical Formula: C_28_H_27_ClN_4_OS^2^**·**^+^. Exact Mass: 502.16. Found: [M + H]^3+^/2: 251.10725, [M + H]^3+^: 501.15829.

### 3.3. Cell Isolation and Culturing

When routinely extracted for orthodontic indications, third molars were immediately transported to the laboratory in phosphate-buffered saline (PBS; Lonza, CA, USA). Apical papilla was gently separated from the mineralized roots using a scalpel, then minced into small pieces (>3 mm^2^) and enzymatically digested in 3 mg/mL collagenase type I and 4 mg/mL dispase (Sigma-Aldrich, St. Louis, MO, USA) for 60 min at 37 °C. After centrifugation at 1500 rpm for 5 min at 4 °C, the supernatant was discarded and the cell pellet was resuspended in DMEM (ThermoFisher Scientific Co., Waltham, MA, USA) supplemented with 1% antibiotic/antimycotic (Invitrogen, Waltham, MA, USA) and 10% heat-inactivated fetal bovine serum (Sigma-Aldrich) and seeded in 2 cm^2^ culture dishes. This study was conducted in accordance with the Declaration of Helsinki. All patients participating in this study signed an informed consent form following the decision of the Ethical Committee of the Medical University, Sofia’s Council of Medical Science (No. 4770\11.12.2018).

The cell culture medium was replaced every second or third day. After obtaining 80% confluency, explants were detached with 0.05% trypsin/ethylenediaminetetraacetic acid (Lonza, Gampel, Switzerland) for 15 min, cells were counted using a hemocytometer, and the total cell number was determined. Cells were transferred to 25 cm^2^ or 75 cm^2^ tissue culture flasks (TPP). Further cultivation was carried out until reaching 80% confluence. For the staining, cells at the fifth passage were seeded in 6-well plates. All experiments were performed in triplicate. Nonstained cells were used as controls.

The study of the properties of **R9** in living cells was carried out with the In Cell Analyzer 6000 (General Electric, Boston, MA, USA), for a high-throughput analysis of fixed and living cells. A *Z*-axis scanning confocal mode was used, allowing the observation of a 3D image and the elimination of unwanted fluorescence background. The laser microscope is equipped with four excitation lasers: 405 nm (diode laser), 488 nm (DPSS), 561 nm (DPSS), and 642 nm (diode laser). Bandpass filters with central wavelengths of 455 (FWHM ~ 50 nm), 525 (FWHM ~ 20 nm), 605 (FWHM ~ 50 nm), and 706 (FWHM ~ 70 nm) nm were used.

## 4. Conclusions

A new asymmetric monomethine cyanine dye with benzothiazolium and chloroquinolinium end groups connected with propylpyridinium and propionamide side functionalities was synthesized. The chemical structure of the new dye was determined by ^1^H-NMR, ^13^C-NMR, and ^13^C-DEPT135 NMR spectroscopy and HPLC-ESI-MS spectrometry. Quantum chemical calculations revealed the charge distribution within the molecule and the intramolecular charge transfer (ICT) character of the lowest-energy electronic transition. The obtained new dye is characterized by high molar absorbance and a strong fluorescence response after the addition of DNA or RNA. The results obtained after measuring the spectra of DNA and RNA solutions by circular dichroism upon titration with the obtained new dye indicated the most likely minor groove nucleic acid binding mechanism. The newly developed dicationic dye presents limitations in permeability, which constrain its functionality as a probe for RNA and DNA detection. To improve cellular delivery, we employed a patented technology referred to as CellInject, which facilitates the efficient introduction of the dye into cells while minimizing its extraneous activity.

Our investigation, conducted on two human cell lines, demonstrated notable enhancements in membrane permeability and increased selectivity, supporting the visualization of nucleic acids within both the cytoplasm and nucleus. Importantly, both the carrier and the dye exhibited biocompatibility and a lack of cytotoxicity. These findings suggest that the **R9** dye in conjunction with CellInject may serve as an effective fluorogenic probe for cellular staining and nucleic acid visualization.

## Figures and Tables

**Table 1:** Absorption maxima ($\lambda_{max}^{abs}$) in eV, and oscillator strength f (in parentheses) for the dye **R9**, computed at TDPBE0/6-31+G(d,p) and TDHFS/6-31+G(d,p) levels in water medium (IEFPCM model).

Dye	$\lambda_{max}^{abs}/\mathrm{eV}$		
	Exp	TDPBE0/6-31+G(d,p)	TDHFS/6-31+G(d,p)
**R9**	2.43 ^a^	2.79 (0.9750)	2.50 (0.8128)

^a^ The experimental value—2.43 eV, 510 nm.

**Table 2:** B3LYP/6-31+G(d,p) calculated NBO, MK, Hirshfeld atomic charges, and EPN values for the nitrogen atoms in the **R9** molecule.

	q_NBO_	q_MK_	q_Hirsh_	EPN
N1	−0.3881	−0.0647	0.0087	−18.0424
N2	−0.3537	−0.0296	0.0289	−18.0671
N3	−0.3041	0.2441	0.0745	−17.9657
N4	−0.8568	−1.0267	−0.1151	−18.1692

**Table 3:** Absorption and fluorescence maxima of dye **R9** neat and in the presence of dsDNA and RNA.

Sample	Abs λ_max_ (nm), (ε (L·mol^−1^·cm^−1^))	Fl λ_max_ (nm)	I_Fl_ (cps)	ΔI = I_Fl_/I_0_
**R9** neat	510 (50,540)	541	1.1 × 10^5^	-
**R9**—dsDNA	518 (37,700)	541	9.4 × 10^6^	85
**R9**—RNA	514 (37,210)	541	1.5 × 10^7^	136

## Data Availability

The original contributions presented in the study are included in the article/Supplementary Materials, further inquiries can be directed to the corresponding authors.

## Supplementary Materials

The following supporting information can be downloaded at https://www.mdpi.com/article/10.3390/molecules29246038/s1, Figure S1. ^1^H-NMR spectra in DMSO-_d6_ of dye 2-((1-(3-amino-3-oxopropyl)-7-chloroquinolin-4(1H)-ylidene)methyl)-3-(3-(pyridin-1-ium-1-yl)propyl)benzo[d]thiazol-3-ium (**R9**); Figure S2. ^13^C-DEPT 135 MHz of dye **R9**; Figure S3. HPLC-ESI-MS spectra of dye **R9**; Figure S4. DLS spectra of pure SLN particles; Figure S5. DLS of SLNP loaded with dye **R9**; Video S1. Control_SLN; Video S2. **CellsSLNP_R9**.
