# Supplementary material for: Nanoconfined Chlorine-Substituted Monomethine Cyanine Dye with a Propionamide Function Based on the Thiazole Orange Scaffold—Use of a Fluorogenic Probe for Cell Staining and Nucleic Acid Visualization
Source: Molecules. 2024 Dec 21;29(24):6038. doi: 10.3390/molecules29246038 (PMC11677322; doi:10.3390/molecules29246038)
Supplement: Supplementary file 1 [file molecules-29-06038-s001.zip › molecules-3287449-supplementary.pdf]

Supplementary Materials

for

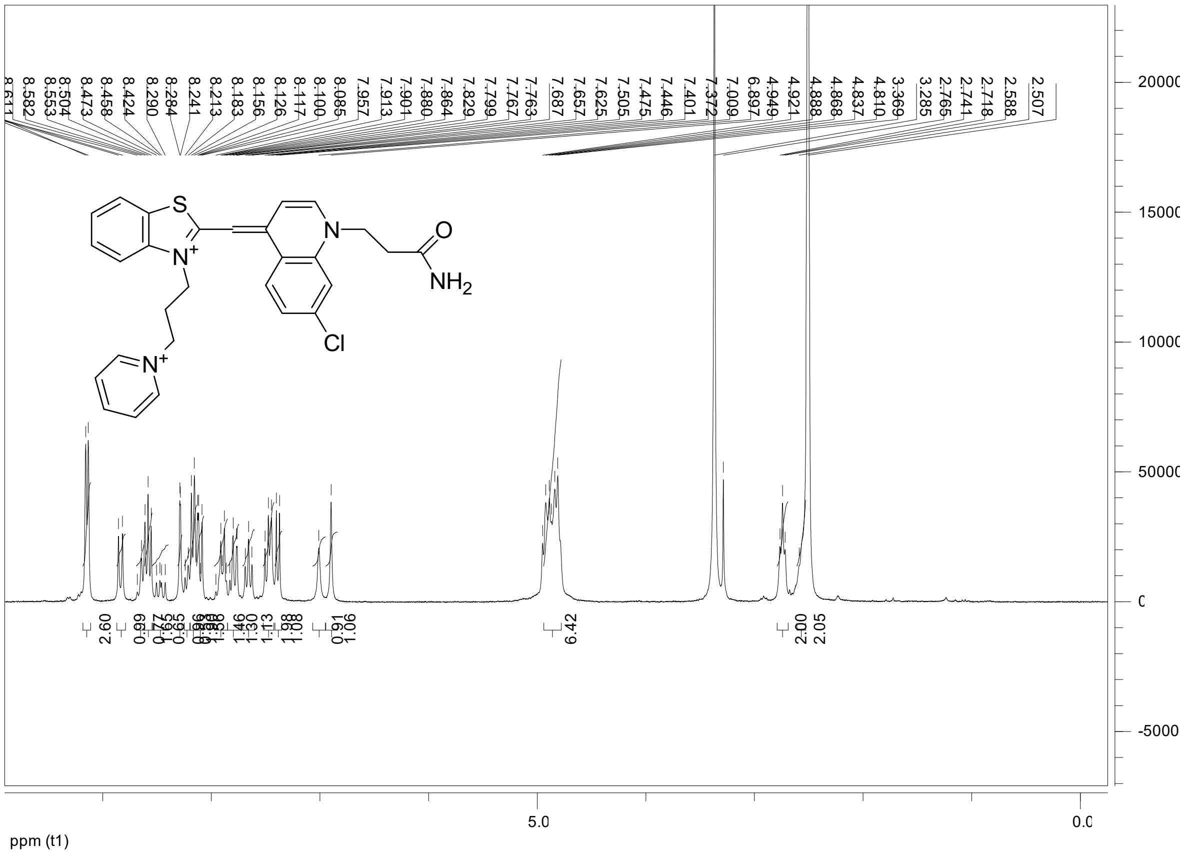

Figure S1. ^{1}H-NMR spectra in DMSO-d_6 of dye 2-((1-(3-amino-3-oxopropyl)-7-chloroquinolin-4(1H)-ylidene)methyl)-3-(3-(pyridin-1-ium-1-yl)propyl)benzo[d]thiazol-3-ium (**R9**).

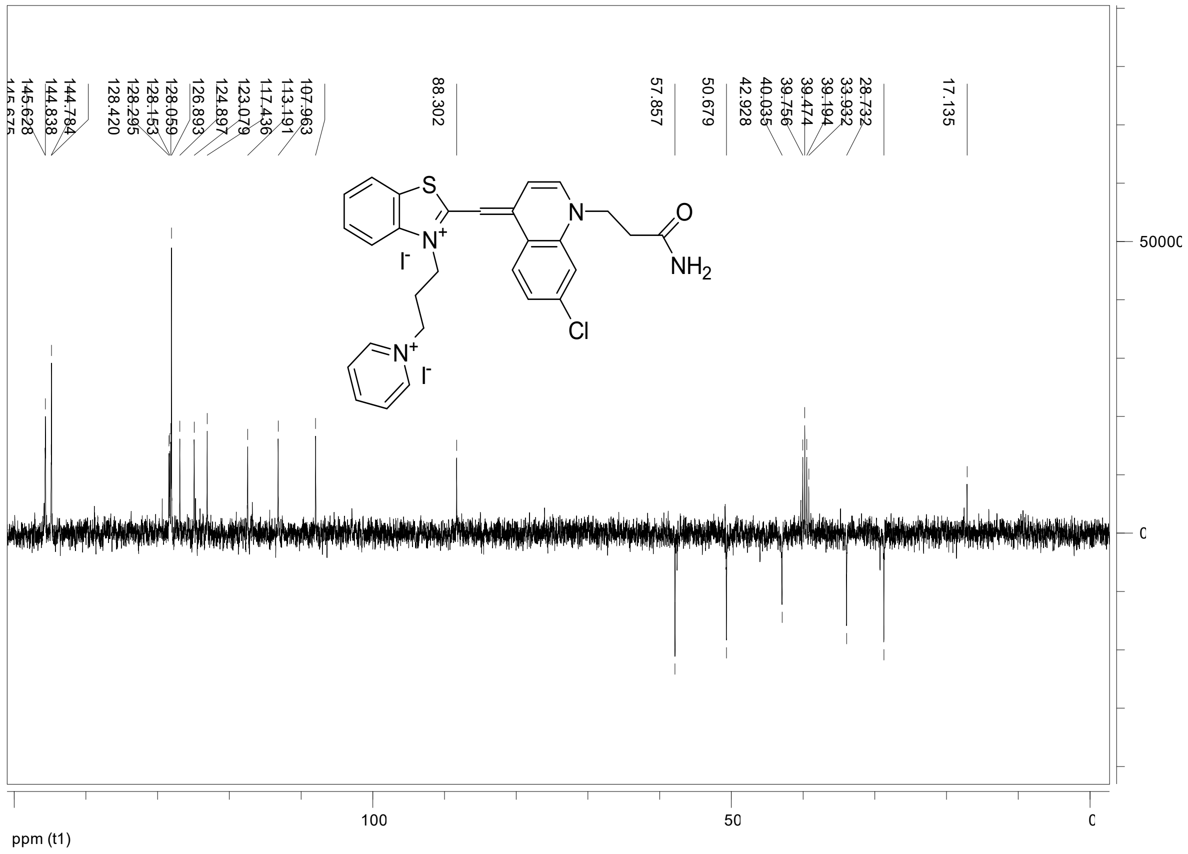

Figure S2. ^{13}C-DEPT 135 of dye **R9**.

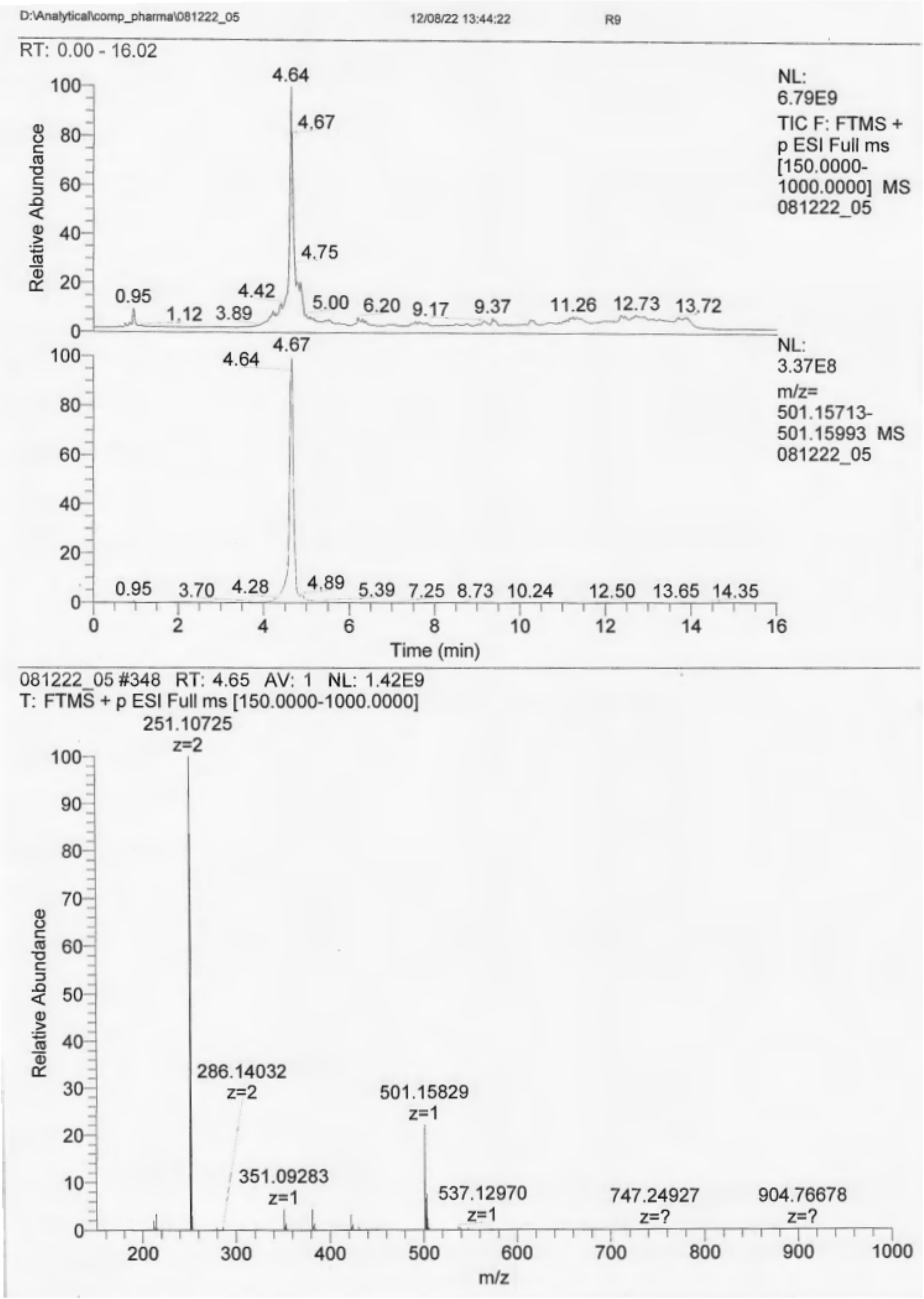

Figure S3. HPLC-ESI-MS spectra of dye R9.

Size Distribution Report by Intensity

v2.2

Malvern

Sample Details

Sample Name: G3 1

SOP Name: mansettings.nano

General Notes:

**File Name:**	Example Results.dts	**Dispersant Name:**	Water
**Record Number:**	97	**Dispersant RI:**	1.330
**Material RI:**	1.44	**Viscosity (cP):**	1.0031
**Material Absorbtion:**	0.001	**Measurement Date and Time:**	Thursday, July 01, 2021 4:1...

System

**Temperature (°C):**	20.0	**Duration Used (s):**	50
**Count Rate (kcps):**	404.9	**Measurement Position (mm):**	4.65
**Cell Description:**	Disposable sizing cuvette	**Attenuator:**	11

Results

			Size (d.n...	% Intensity:	St Dev (d.n...
**Z-Average (d.nm):**	37.50	**Peak 1:**	41.15	98.9	14.10
**PdI:**	0.141	**Peak 2:**	4765	1.1	738.9
**Intercept:**	0.943	**Peak 3:**	0.000	0.0	0.000

Result quality Good

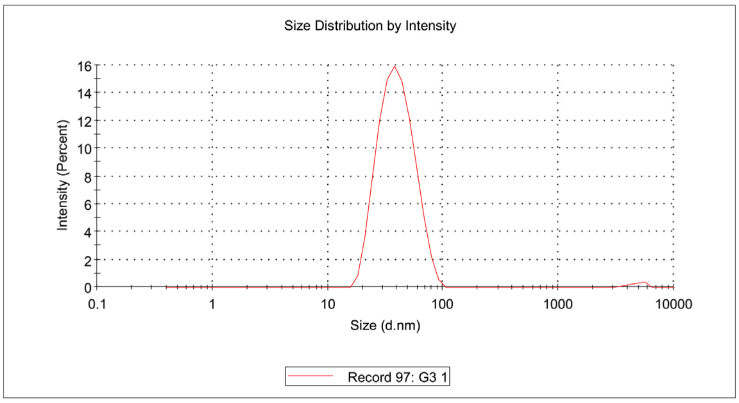

Figure S4. DLS spectra of pure SLN particles.

Size Distribution Report by Intensity

v2.2

Malvern

Sample Details

Sample Name: G9 1

SOP Name: mansettings.nano

General Notes:

**File Name:**	Example Results.dts	**Dispersant Name:**	Water
**Record Number:**	98	**Dispersant RI:**	1.330
**Material RI:**	1.44	**Viscosity (cP):**	1.0031
**Material Absorbtion:**	0.001	**Measurement Date and Time:**	Thursday, July 01, 2021 4:2...

System

**Temperature (°C):**	20.0	**Duration Used (s):**	50
**Count Rate (kcps):**	140.4	**Measurement Position (mm):**	4.65
**Cell Description:**	Disposable sizing cuvette	**Attenuator:**	10

Results

			Size (d.n...	% Intensity:	St Dev (d.n...
**Z-Average (d.nm):**	37.58	**Peak 1:**	40.72	99.0	12.93
**PdI:**	0.145	**Peak 2:**	4900	1.0	668.5
**Intercept:**	0.973	**Peak 3:**	0.000	0.0	0.000

Result quality Good

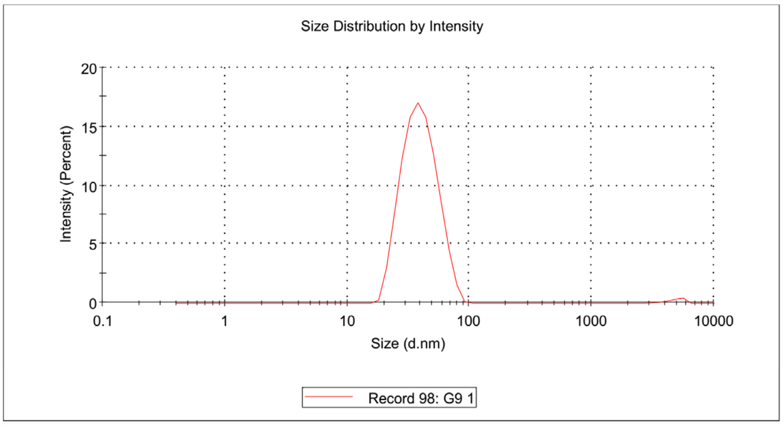

Figure S5. DLS of SLNP loaded with dye **R9**.
